# Comprehensive Mapping of Regional Expression of the Clock Protein PERIOD2 in Rat Forebrain across the 24-h Day

**DOI:** 10.1371/journal.pone.0076391

**Published:** 2013-10-04

**Authors:** Valerie L. Harbour, Yuval Weigl, Barry Robinson, Shimon Amir

**Affiliations:** Center for Studies in Behavioral Neurobiology, Department of Psychology, Concordia University, Montreal, Quebec, Canada; Hôpital du Sacré-Coeur de Montréal, Canada

## Abstract

In mammals, a light-entrainable clock located in the suprachiasmatic nucleus (SCN) regulates circadian rhythms by synchronizing oscillators throughout the brain and body. Notably, the nature of the relation between the SCN clock and subordinate oscillators in the rest of the brain is not well defined. We performed a high temporal resolution analysis of the expression of the circadian clock protein PERIOD2 (PER2) in the rat forebrain to characterize the distribution, amplitude and phase of PER2 rhythms across different regions. Eighty-four LEW/Crl male rats were entrained to a 12-h: 12-h light/dark cycle, and subsequently perfused every 30 min across the 24-h day for a total of 48 time-points. PER2 expression was assessed with immunohistochemistry and analyzed using automated cell counts. We report the presence of PER2 expression in 20 forebrain areas important for a wide range of motivated and appetitive behaviors including the SCN, bed nucleus, and several regions of the amygdala, hippocampus, striatum, and cortex. Eighteen areas displayed significant PER2 rhythms, which peaked at different times of day. Our data demonstrate a previously uncharacterized regional distribution of rhythms of a clock protein expression in the brain that provides a sound basis for future studies of circadian clock function in animal models of disease.

## Introduction

Circadian rhythms establish the timing of biological systems in order to optimize physiology, behavior and health [1], [2]. In mammals, these rhythms are generated by a network of cellular clocks scattered throughout the brain and periphery, and governed by a master pacemaker located in the suprachiasmatic nucleus of the hypothalamus, SCN [3]. Both the anatomical connections and cellular organization of the SCN pacemaker and the distribution of circadian clocks in the periphery have been the focus of intense investigation, whereas, a similar in-depth investigation of the properties of the network of circadian clocks in the brain is lacking.

The Period2 (PER2) protein is a core constituent of the mammalian circadian clock, and the rhythmic expression of PER2 has been widely used as a marker of clock cells in both neural and non-neural tissues in rodents [4]–[7]. Previous studies on the expression of clock genes in the rodent brain have identified circadian oscillations in the SCN and in a number of other intrinsically rhythmic neural structures, including the retina and olfactory bulb [8], [9]. However, daily rhythms in the expression of PER2 and other clock genes and proteins have been seen in many other neural structures that are not considered intrinsically rhythmic, suggesting that many brain nuclei harbor functional circadian clocks [10], [11]. Consistent with this suggestion, we have identified robust daily rhythms in expression of PER2 in five functionally and anatomically interconnected regions of the rat limbic forebrain, the oval nucleus of the bed nucleus of the stria terminalis (BNSTov), the lateral part of the central amygdala (CEAl), the basolateral amygdala (BLA), the dentate gyrus (DG) of the hippocampus, and the dorsal striatum [12]–[15]. Significantly, we also found that the PER2 rhythms in the BNSTov and CEAl, nuclei which form a distinct functional unit known as the central extended amygdala [16], peaked in the evening, in phase with the rhythm in the SCN. In contrast, the rhythms found in the BLA, DG, and striatum peaked at the opposite time of day, in the morning, revealing distinct phase relationships between PER2 oscillations in different forebrain nuclei and the PER2 rhythm in the SCN.

Most previous studies on the distribution and rhythmic expression of clock genes and proteins in the rodent brain have used only a few time-points for the analysis, resulting in low temporal resolution and loss of important information on the true phase and amplitude of region-specific rhythms. In the present study, we analyzed the expression of PER2 in 20 forebrain areas collected every 30 min throughout the 24-h day in order to obtain more precise and detailed information about the phase and amplitude of PER2 oscillations in the brain. Our specific goals were to re-examine and refine PER2 expression patterns in the SCN, BNSTov, CEAl, BLA, DG, and striatum across more time-points; to characterize the patterns of PER2 expression in additional subregions of the amygdala, hippocampus, striatum, and in the cortex; and to establish the phase relationships between all rhythmic forebrain regions and between these regions and the SCN. The ensuing atlas presents a fine grain analysis of PER2 oscillations in the forebrain and provides a much-needed foundation for studying the regulation and function of circadian clocks in anatomically defined regions of the brain.

## Methods

### Animals and Housing

Eighty-four inbred male Lewis (LEW/Crl) rats weighing 150–200 g upon arrival (Charles River, St-Constant, QC) were used. Rats arrived in seven successive batches of 12 with each batch housed in the same experimentation room. Rats were individually housed in cages (9.5 in wide×8 in height×16 in deep) equipped with running wheels and had *ad libitum* access to rat chow and water. Each cage was housed within a custom-built ventilated, sound and light-tight isolation chamber (17.5 in wide×27.5 in height×27.5 in deep) equipped with a computer-controlled lighting system (VitalView software; Mini Mitter Co. Inc., Sunriver, OR). Wheel-running activity (WRA) was recorded continuously and displayed in 10-min bins using VitalView software. Actograms were then created and analyzed to verify stable entrainment to the light/dark (LD) cycle using Circadia software (v2.1.6). All procedures were carried out in accordance with the Canadian Council on Animal Care guidelines and were approved by the Animal Care Committee of Concordia University.

### Procedure

Rats were kept on one of four 12-h: 12-h LD cycles (lights on at 3∶00, 9∶00, 15∶00, or 21∶00; 100 lux at cage bottom) to allow perfusions to be done during working hours. All rats entrained to their LD cycle with the majority of the WRA being confined to the night (or dark) portion of the cycle. After three to four weeks rats were perfused every 30 min across the 24-h day to give a total of 48 *zeitgeber* times (ZT; where ZT0 denotes lights on and ZT12 denotes lights off) with 1–3 rats per time-point, except for ZT1 and ZT13, which have 4 rats per group. Within each of the seven groups, rats were assigned perfusion times spread across the 48 time-points in order to have a representative distribution of ZTs in each group and to control for potential inter-assay differences.

### Tissue Preparation

At the appropriate time, rats were deeply anaesthetized with sodium pentobarbital (∼100 mg/kg, i.p.) and perfused transcardially with 300 ml of cold saline (0.9% NaCl) followed by 300 ml of cold paraformaldehyde (4% in a 0.1 M phosphate buffer, pH 7.3). Brains were removed and post-fixed overnight in paraformaldehyde at 4°C. Serial coronal sections (50 µm thick) through regions of interest were collected using a Vibratome tissue slicer (St-Louis, MO) and stored in Watson’s Cryoprotectant [17] at –20°C until processed for immunohistochemistry (IHC).

### Immunohistochemistry

IHC for PER2 was carried out in six independent runs within a two-week time frame, each containing brain sections from rats perfused at times spread across the 48 time-points. This ensured that any minor differences in staining between runs would be randomized across the time-points and rendered obsolete. In each run, free-floating sections were rinsed (6×10 min) in cold 0.9% Trizma buffered saline (TBS; pH 7.6) and incubated in a hydrogen peroxide quench solution (3% H_2_O_2_ in TBS) for 30 min at room temperature. Sections were then rinsed (3×10 min) in TBS and incubated in a pre-block solution made of 0.3% Triton X-100 in TBS (Triton-TBS) and 5% Normal Goat Serum (NGS), for 1-h at 4°C, and then directly transferred into the primary solution. Sections were incubated in PER2 polyclonal antibody (Alpha Diagnostic International, San Antonio, TX; see Table 1) diluted 1∶800 in Triton-TBS and 3% NGS, and incubated for approximately 48-h at 4°C. After the primary antibody incubation, sections were once again rinsed in cold TBS and then transferred to a secondary solution consisting of biotinylated anti-rabbit IgG made in goat (Vector Laboratories, Burlington, ON) diluted 1∶200 in Triton-TBS and 3% NGS for 1-h at 4°C. After incubation with the secondary antibody, sections were rinsed (3×10 min) with cold TBS and incubated in a tertiary solution (avidin biotin peroxidase complex in TBS) for 2-h at 4°C (Vectastain Elite ABC Kit, Vector Laboratories). Finally, sections were rinsed in TBS, and then again in cold 50 mM Tris-HCl for 10 min. Sections were then incubated for 10 min in 0.05% 3,3′-diaminobenzidine (DAB) in Tris-HCl and further incubated for 10 min in DAB/50 mM Tris-HCl with 0.01% H_2_0_2_ and 600 µl of 8% NiCl_2_ solution per 150 ml of DAB solution. Sections were rinsed a final time in cold TBS and wet-mounted onto gel-coated microscope slides, allowed to dry overnight, and then dehydrated through graded ethanol concentrations, soaked in Citrisolve (Fisher Scientific, Houston, TX) for a minimum of 30 min, and finally cover-slipped with Permount (Fisher).

**Table 1:** Primary antibody used.

Antigen	Immunogen	Manufacturer, species, type	Dilution used
PERIOD2	23-AA peptide sequence (CDTSEA KEEEGEQLTG PRIEAQT)corresponding to AA 1235–1257 of mouse PER2 (epitope locationC-terminus, gene accession no. 054943) conjugated to KLH(Cat # PER21-P)	ADI (San Antonio) Rabbit polyclonal, affinitypure IgG; (Cat # PER21-A)	1∶800

### Antibody Characterization

**Figure 1:**
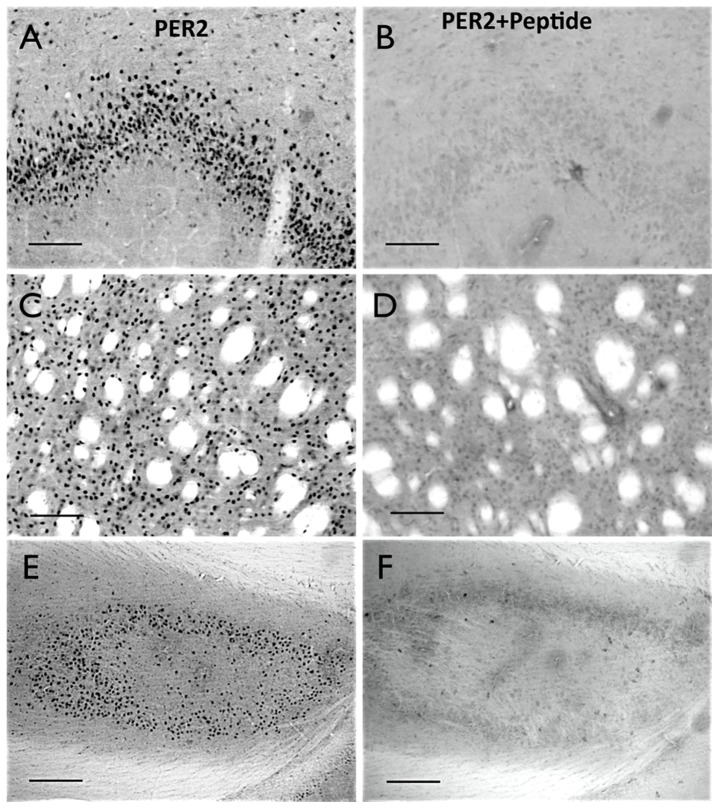
Blocking experiment. Representative photomicrographs of the piriform cortex (**A, B**), striatum (**C, D**), and hippocampus (**E, F**), stained for PER2 without (**A, C, E**) and with (**B, D, C**) the PER2 peptide. Scale bar 100 µm for the piriform and striatum; 200 µm for the hippocampus.

The PER2 antibody used in this study is a polyclonal affinity pure IgG raised in rabbit against a 23 amino acid peptide corresponding to amino acids 1235–1257 of mouse PER2 (CDTSEA KEEEGEQLTG PRIEAQT, epitope location: C-terminus, gene accession # 054943). This antibody has been validated and used extensively in previous IHC analysis of PER2 in mouse and rat brain [13], [15], [18]–[24]. We further confirmed the specificity of the PER2 antibody in blocking experiments using the IHC procedure described above except that the PER2 peptide, reconstituted in 1 mg/ml in PBS, pH 7.4, with 0.02% sodium merthiolate diluted 1∶100, was added to the primary incubation solution. This manipulation prevented PER2 staining. Representative photomicrographs of PER2 staining in brain sections from the DG, piriform cortex and dorsal striatum with and without the added peptide are shown in Fig. 1.

### Data Analysis

**Figure 2:**
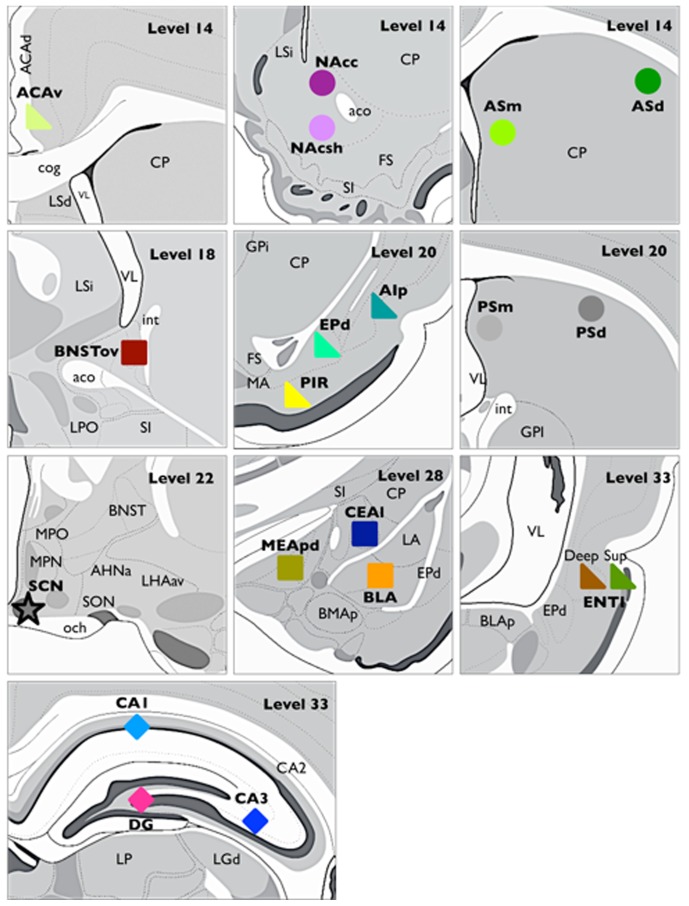
Brain regions analyzed. Schematic diagrams depicting the location of each brain region analyzed, levels correspond to location in Swanson atlas. Five major areas were analyzed: SCN 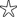, Amygdala □, Hippocampus ⋄, Striatum ○, and Cortex 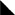, with a total of 20 sub-regions.

Brain sections were examined under a light microscope using a 20X objective (Leitz Laborlux S). General inspection of brain sections revealed equally high quality (intensity, background) of PER2 immunostaining in different brain regions within and across different IHC runs. Regions of interest were identified using Brain Maps: Structure of the rat brain 3rd edition [25], except for the nucleus accumbens, where The Rat Brain 5th edition [26] was used. Given the large size of the dorsal striatum, we determined that a simplified version of Willuhn et al.’s striatal subdivisions [27] was the most appropriate to use in our context, due the potential significance of the various afferent projections from different nuclei on the function of local clocks. We examined four areas of the striatum based on distinct cortical afferents: a medial division, adjacent to the lateral ventricles, was recognized due to distinct afferents from both the ventral and dorsal anterior cingulate cortex. A dorsal division, located laterally just under the corpus callosum, was recognized due to afferents from the somatosensory cortex and medial agranular cortex (corticostriatal afferents are reviewed in [27]). Moreover, due to such a large rostral-caudal expansion of the striatum, these regions were further subdivided into anterior and posterior sections, with the joining of the anterior commissure as the boundary between the anterior and posterior portions, yielding a total of four striatal subdivisions (see Fig. 2).

Before capturing a new stack of images for a given region and rat, the contrast for the camera was enhanced once while focusing on a region of the cortex containing PER2-immunoreactive (PER2-ir) cells, thereby ensuring that similar camera settings were used. Once identified, all regions were digitized using a Sony XC-77 Video Camera, a Scion LG-3 frame grabber, and NIH Image software (v1.63, http://rsb.info.nih.gov/nih-image/). Multiple images from each region were captured using a 400×400 µm template. Cells immunopositive for PER2 were counted by an observer blind to group membership (in this case ZT) using Image SXM software (v1.9, SD Barrett, http://www.ImageSXM.org.uk). Using this program, images from regions where PER2-ir cells are tightly clustered (e.g. SCN and DG) were sharpened and smoothed twice. To determine immunopositive cells, particle analysis was set to recognize nuclei between 9 and 99 pixels. This range allows for the elimination of pixelated artifacts (dots) that would be too small to be stained nuclei while slightly underestimating the size of unusually large stained nuclei. The pixel intensity threshold was set to highlight all visible cells within each brain region, and each region was analyzed independently. The Abercrombie equation [28] was used to obtain unbiased estimates of the number of PER2-labeled nuclei, with the corrected count being N = n×T/(D+T), where n is the raw cell count, T is the thickness of the tissue section (50 µm) and D is the mean diameter of stained nuclei (9.5 µm, based on measurements of 100 nuclei selected randomly from different regions of interest from four randomly selected rats). For each of the 84 rats, the corrected counts from the five unilateral sections containing the highest number of labelled nuclei in each region were averaged and the means were used in the statistical analysis.

### Statistics

All analyses and graphs were done using GraphPad Prism (v5.0, HJ Motulsky, www.graphpad.com/prism). Data points for each individual rat were plotted for each brain region with number of PER-ir cells on the Y-axis and ZT on the X-axis, thus, each data point (diamond) in the sine wave graphs is that of a single rat. Sine waves (least-squares regression) were then fit to each graph in order to better visualize time of peak and trough for each region using the following equation: Y = M+A*sin (F*X+PS); where M stands for the mesor (i.e. average of the spread of the data from the mid-point of the curve), A stands for amplitude (calculated from the mesor), F stands for frequency (in radians), and PS stands for phase shift, which is the earliest time Y = 0 measured in X axis units. Outliers were determined using the ROUT method with a False Discovery Rate of 1% and removed from all analyses (see [29] for details of the procedure). Outliers are identified in the graphs by a red diamond. A sine wave with the frequency constrained to exactly 24-h was then fitted to the data. To further assess the goodness of fit of the curves generated from the sine wave model, the D’Agostino-Pearson omnibus K^2^ normality test [30] was applied to the data. The normality test examines skewness and kurtosis in order to assess how far from Gaussian the data are. A data set fails the normality test when the *p* value is ≤0.05, meaning that it deviates significantly from a normal distribution. The statistics for this test are mentioned in the Results section only if they are significant (indicating that the data set has failed the test).

In addition to the tests mentioned above, one-way analyses of variance (ANOVA) were used to compare the amplitudes of PER2 rhythms (established by the sine-fitting model, measured in PER2-ir cells from peak to trough) in different sub-regions of each broader anatomical area (for example, the DG, CA1, and CA3 of the hippocampus). Significant effects were further analyzed with Bonferroni multiple comparison post-hoc tests. In order to compare the strength of the rhythm in each region, we derived a Rhythmicity Index (RI) value by dividing the peak to trough amplitude by the mesor (the average of the spread of the data). Rhythmicity Indices for each region were then normalized to the SCN, which was given a value of 1.0. The RI therefore takes into account the amplitude of the PER2 rhythm as well as the average PER2 expression, providing a more meaningful analysis of the rhythms, and allowing for comparisons of rhythmicity between regions with different levels of PER2 expression.

Because the SCN shows the most robust rhythm in PER2 expression in the brain, the fit (R^2^ value) from the sine wave model and the normalized amplitude (Rhythmicity Index value) for the SCN were used as the standard for determining the rhythmicity criteria for other regions. Specifically, for the PER2 data, a brain region was considered rhythmic if the R^2^ value was 1/3rd or more of that of the SCN and the RI value was at least 25%. These criteria were selected to enable the identification of rhythmic areas, while excluding amplitudes that would be too low to ascertain rhythmicity with some degree of confidence. Finally, in order to obtain statistical evidence of relationships between the rhythms of PER2 expression in different regions, correlations (Pearson r) were calculated using individual data points from each rat. Alpha level was set to 0.05 for all analyses.

## Results

We used IHC to characterize the patterns of expression of PER2-ir in the SCN, amygdala, hippocampus, striatum, and cortex of rats that were housed under a normal LD cycle. In total, 20 regions were analyzed every 30 min throughout the 24-h day. Notably, the estimates of PER2-ir cells used in the different analyses were based on automated counts from selected brain sections and not on unbiased stereological methods. Therefore, these estimates could reflect local levels that may not necessarily be representative of the whole region sampled. Fig. 2 shows schematic diagrams illustrating the regions examined and their respective location.

### SCN

**Figure 3:**
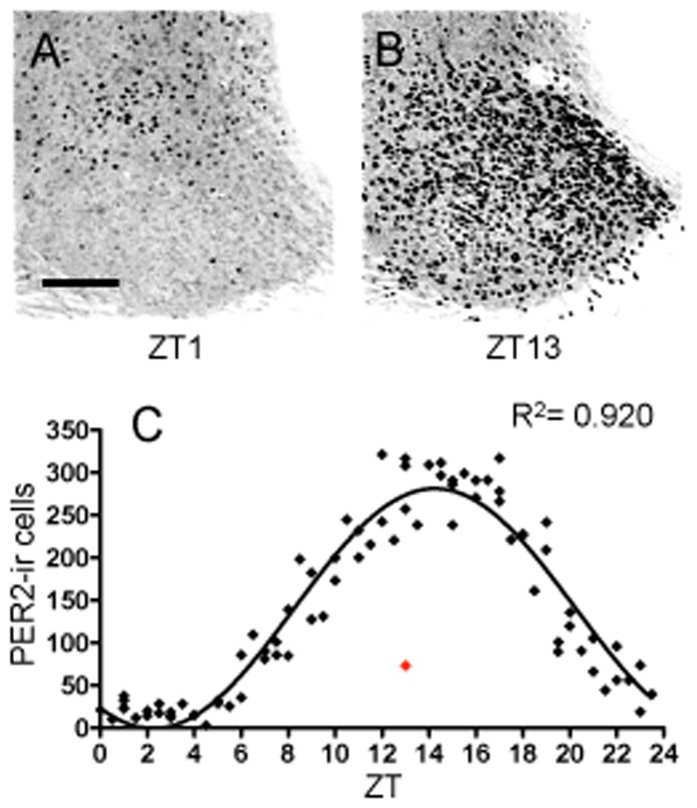
Suprachiasmatic nucleus. (**A–B**) Representative photomicrographs of PER2-ir cells in the SCN at ZT1 and ZT13, scale bar 100 µm. (**C**). Mean number of PER2-ir cells in the SCN of each individual rat (diamonds) across 48 *zeitgeber* times fitted with a 24-h sine wave. R^2^ = Goodness of fit value (*n* = 81, outlier = red diamond).

**Figure 4:**
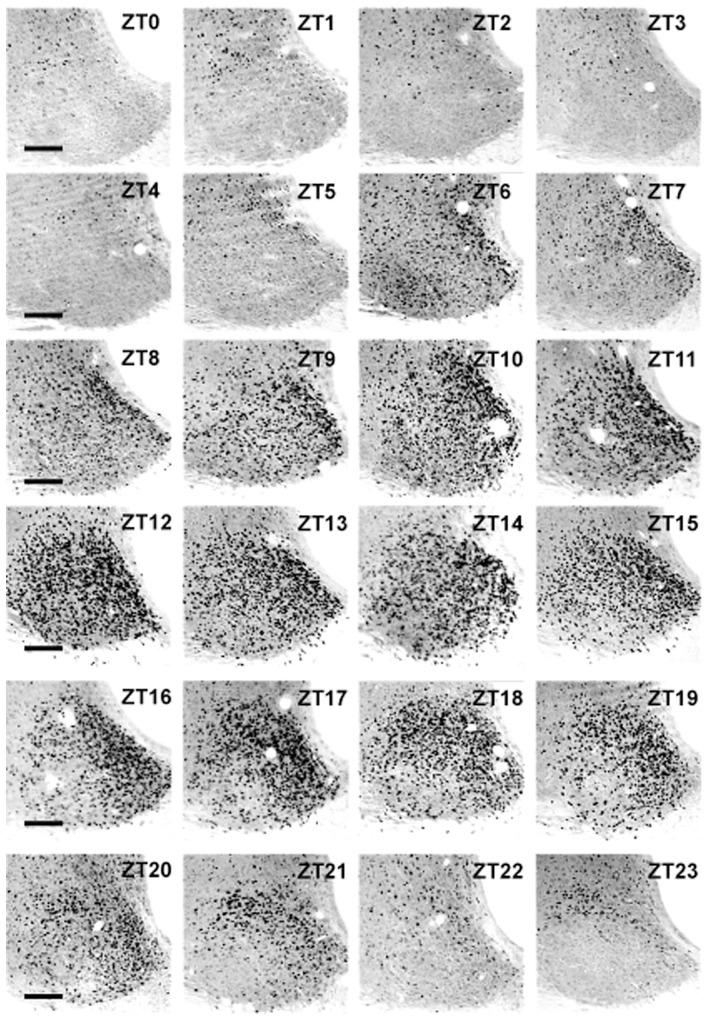
PER2 in the SCN. Representative photomicrographs of PER2-ir cells in the SCN every hour across the 24-h day. *Zeitgeber* (ZT) time 0 denotes lights on, ZT12 lights off. Scale bar 100 µm.

PER2 expression in the SCN followed a high amplitude (peak to trough value of 281.8 immunoreactive cells) daily rhythm with peak expression of PER-ir cells occurring in the early part of the dark phase, at ZT14.25 (Fig. 3), slightly later than previously found when using fewer time-points [13], [19], [20]. The 24-h sine wave model for the SCN fit a curve with an R^2^ value of 0.920, therefore, a brain region must have an R^2^ value of at least 0.307 (1/3rd of that of the SCN) to meet the criteria for rhythmicity. Photomicrographs showing examples of PER2-ir cells in the SCN every hour across the 24-h day are shown in Fig. 4.

### BNSTov and Amygdala

The BNSTov and CEAl are neurochemically homologous and anatomically interconnected regions of the limbic forebrain that form a functional unit known as the central extended amygdala [16], [31], [32]. The BNSTov and CEAl play important roles in the neuroendocrine and autonomic responses to stress have been shown to be involved in diverse behaviors, such as feeding [33], drug use and relapse [34]–[36], and fear and anxiety [37], [38]. The BLA resembles the neighbouring cortex more than the rest of the amygdala, and projects mainly to the medial CEA, the cortex, and the hippocampus through the entorhinal cortex (ENT; [39]–[41]), but does not have dense connections to the BNSTov [39].

#### PER2 expression in the BNSTov and CEAl

**Figure 5:**
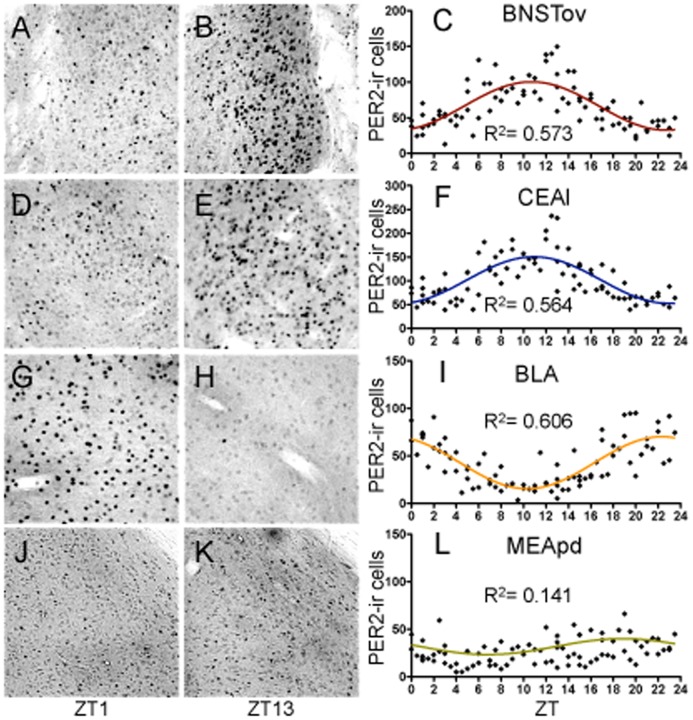
Amygdala and bed nucleus of the stria terminalis. Representative photomicrographs of PER2-ir cells in the oval nucleus of the BNST (**A–B**), central amygdala, lateral part (**D–E**), basolateral amygdala (**G–H**), and medial amygdala, posterior dorsal part (**J–K**) at ZT1 and ZT13, scale bar 100 µm. Mean number of PER2-immunoreactive cells in the BNSTov (**C**), CEAl (**F**), BLA (**I**), and MEApd (**L**) of each individual rat (diamonds) across 48 *zeitgeber* times fitted with a 24-h sine wave. R^2^ = Goodness of fit value for given region. *n* = 84 for all regions.

The BNSTov showed a highly rhythmic pattern of PER2 expression, with an R^2^ value of 0.573 and amplitude of 67.68 (Fig. 5A–C). PER2 expression in the BNSTov peaked at approximately ZT11, slightly earlier than previously reported when using fewer time-points [13]. Similarly, PER2 expression in the CEAl was strongly rhythmic (R^2^ = 0.564) with amplitude of 97.86 (Fig. 5D–F). As previously reported [15], PER2 expression in the CEAl is in perfect phase with that of the BNSTov, peaking at ZT11.

#### PER2 expression in the BLA

PER2 expression in the BLA was highly rhythmic (R^2^ = 0.606) with amplitude of 54.72 (Fig. 5G–I). Expression in the BLA peaked at the end of the dark phase, around ZT22.5, and, significantly, was in anti-phase with the rhythms seen in the BNSTov and CEAl, as previously reported [15].

#### PER2 expression in the medial amygdala, posterior dorsal (MEApd)

PER2 expression in the MEApd was evenly distributed throughout the 24-h day showing an R^2^ of 0.141 and amplitude of 16.74 (Fig. 5J–L). Based on these findings, the PER2 expression profile in the MEApd was deemed arrhythmic, at least under the present experimental conditions.

#### BNSTov and Amygdala: Summary

**Figure 6:**
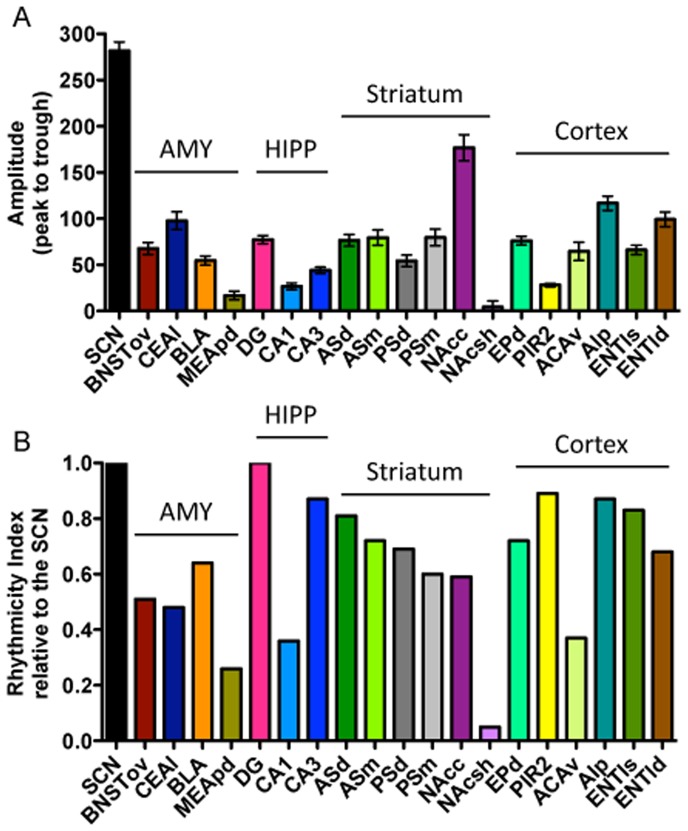
Summary: Comparing amplitude and strength of rhythms between all regions. **A**) Amplitudes (mean number ± SEM of PER2-ir cells) measured from peak to trough, in all brain regions. **B**) Rhythmicity index values for each brain region normalized to the SCN.

It was found that the rhythms of PER2 expression in the BNSTov and CEAl are in phase with each other, while the BLA is in anti-phase with these regions, and the MEApd is arrhythmic. Notably, none of these rhythms is either in phase or in anti-phase with the PER2 rhythm of the SCN. Furthermore, there are significant differences in amplitude between the SCN, BNSTov, and amygdala (*F*_(4,412)_ = 198.60, *p*<0.0001), with the amplitude in the CEAl being higher than in the other sub-regions of the amygdala (BNSTov: *t*_(415)_ = 2.94, *p*<.05, BLA: *t*_(415)_ = 4.20, *p*<.001, MEApd: *t*_(415)_ = 7.90, *p*<.001), and the MEApd being significantly lower (BNSTov: *t*_(415)_ = 4.96, *p*<.001, BLA: *t*_(415)_ = 3.70, *p*<.01, Fig. 6A). Notably, the amplitudes in all the BNST/amygdalar sub-regions are significantly lower than that in the SCN. However, when normalized to take into account the spread of the data for each region relative to the SCN, the BNSTov, CEAl, and BLA show rhythms that are at least 50% as rhythmic as the SCN (RI values of 0.51, 0.50, 0.64, respectively, Fig. 6B), whereas the arrhythmic MEApd has an RI value of only 0.26. Correlations of PER2 expression between the three rhythmic BNST/amygdala regions and the SCN revealed statistically significant moderate relationships (see Table 2). Notably, a high correlation of 0.88 was found between the rhythms in the BNSTov and CEAl, consistent with the evidence that these two regions are highly interconnected and functionally related.

**Table 2:** Correlations of PER2 expressionin the amygdala.

Region	BNSTov	CEAl	BLA
SCN	0.45	0.49	−0.34
BNSTov		0.88	−0.49
CEAl			−0.45

Pearson r values comparing regions of the amygdala and the SCN across the 24-h day. Values in bold are statistically significant (*p*<0.05). The MEApd has been omitted since it is arrhythmic.

### Hippocampus

The hippocampus plays important roles in the neuroendocrine response to stress, in affective disorders, and in learning and memory [42]–[44]. It receives major input from the ENT cortex via the perforant path [45], [46]. The majority of information within the hippocampus flows from the DG, to the CA3, to the CA1. The CA1 acts as the major output of the hippocampus proper sending efferent signals to the ENT cortex and to multiple other cortical regions, including the agranular insular cortex (AI), as well as the thalamus, hypothalamus, and the amygdala [41], [47], [48]. Many of these areas also project back to the hippocampus [49], [50].

#### PER2 expression in the DG, CA1 and CA3

**Figure 7:**
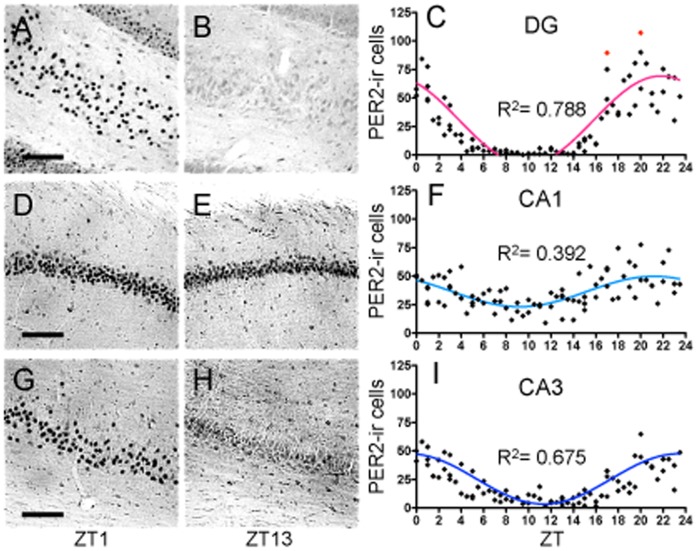
Hippocampus. Representative photomicrographs of PER2-ir cells in the dentate gyrus (**A–B**), CA1 (**D–E**), and CA3 (**G–H**) at ZT1 and ZT13, scale bar 100 µm. Mean number of PER2-immunoreactive cells in the DG (**C**), CA1 (**F**), and CA3 (**I**) of each individual rat (diamonds) across 48 *zeitgeber* times fitted with a 24-h sine wave. R^2^ = Goodness of fit value for given region. *n* = 82 for DG, *n* = 84 for CA1 and CA3 (C: outliers = red diamonds).

The DG showed highly rhythmic PER2 expression (R^2^ = 0.788) with amplitude of 77.26 and peak at approximately ZT22 (Fig. 7A–C). Similarly, the CA3 displays a strong rhythmic pattern in PER2 expression (R^2^ = 0.675, Fig. 7G–I) with amplitude of 44.06 and peak at ZT23.5. In contrast, we found that the CA1 exhibits a relatively weak rhythm in PER2 expression (R^2^ = 0.392) with amplitude of 26.62 (Fig. 7D–F). The peak of PER2 expression in the CA1 occurs at approximately ZT21.5, 30 min earlier than the peak found in the DG and 2-h before the peak in the CA3.

#### Hippocampus: Summary

PER2 expression in the three sub-regions of the hippocampus differs slightly in phase and amplitude (SCN included: *F*_(3,327)_ = 422.60, *p*<.0001). Specifically, the amplitude in the DG is significantly higher than in the CA1 (*t*_(329)_ = 6.28, *p*<.001) and CA3 (*t*_(329)_ = 4.12, *p*<.001), and the PER2 amplitude in the SCN is significantly higher than in all three hippocampal regions (Fig. 6A). Interestingly, calculation of the RI showed that the rhythm in the DG is as strong as the rhythm in the SCN (RI value of 1) and that the CA3 has a very strong rhythm with an RI value of 0.83 (Fig. 6B). The CA1, in contrast, has a relatively weak rhythm compared to the SCN (RI value of 0.36). Correlations between PER2 expression in these hippocampal regions and the SCN revealed differences in the strength of the relationships (for all results consult Table 3). Notably, the rhythm in the DG is moderately correlated with the CA1 (r = 0.60) and highly positively correlated with the CA3 (r = 0.79), and the CA1 and CA3 are moderately correlated (r = 0.54).

**Table 3:** Correlations of PER2 expression in the hippocampus.

Region	DG	CA1	CA3
**SCN**	**−0.33**	**−**0.07	**−0.57**
**DG**		**0.60**	**0.79**
**CA1**			**0.54**

Pearson r values comparing regions of the hippocampus and the SCN across the 24-h day. Values in bold are statistically significant (*p*<0.05).

### Striatum

The striatum plays important roles in motor control, reward, and learning [51]. It consists of the dorsal striatum (also known as caudate putamen), and the ventral striatum, which can be divided into the nucleus accumbens (NAc) core and shell. Four subdivisions of the dorsal striatum were examined (anterior dorsal, anterior medial, posterior dorsal, and posterior medial) because of their distinct cortical afferent projections.

#### PER2 expression in the dorsal striatum

**Figure 8:**
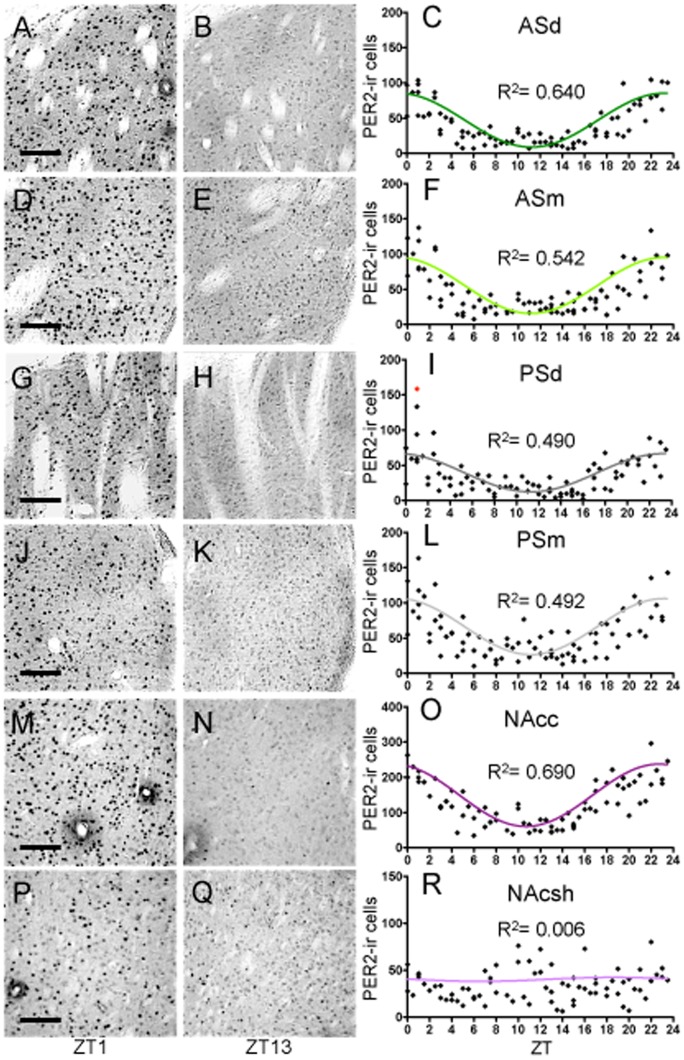
Striatum. Representative photomicrographs of PER2-ir cells in the anterior striatum (AS) dorsal (**A–B**), AS medial (**D–E**), posterior striatum (PS) dorsal (**G–H**), PS medial (**J–K**), nucleus accumbens (NAc) core (**M–N**), and NAc shell (**P–Q**) at ZT1 and ZT13, scale bar 100 µm. Mean number of PER2-ir cells in the ASd (**C**), ASm (**F**), PSd (**I**), PSm (**L**), NAcc (**O**), and NAcsh (**R**) of each individual rat (diamonds) across 48 *zeitgeber* times fitted with a 24-h sine wave. R^2^ = Goodness of fit value for given region. *n* = 84 for ASd, *n* = 83 for PSd, *n* = 82 for ASm and PSm, *n* = 74 for the NAc core and shell (I: outlier = red diamond).

Both the dorsal and medial anterior striatum exhibited rhythmic patterns of PER2 expression (R^2^ = 0.640 and 0.542, respectively; Fig. 8A–F). The PER2 rhythms in the dorsal and medial striatum had similar amplitudes (76.54 and 79.44, respectively) and were in phase, with peak expression occurring at ZT23.5 for each region. Similarly, the dorsal and medial regions of the posterior striatum exhibited rhythmic patterns of PER2 expression (R^2^ = 0.490 and 0.492, respectively; Fig. 8G–L). However, the 24-h model did not fit the data perfectly for the medial part as indicated by a significant normality test for this region (K^2^ = 6.59, *p*<0.05). The posterior dorsal striatum had slightly lower amplitude compared to the medial striatum (54.28 vs. 79.58), however this difference was not statistically significant (*t*_(558)_ = 2.03, *p*>0.05). Importantly, we found that the two subdivisions of the posterior striatum are in phase with each other as well as with the two subdivisions of the anterior striatum, with peak PER2 expression occurring at around ZT23.5.

#### PER2 expression in nucleus accumbens (NAc)

We found that the core and shell of the NAc showed strikingly different patterns of PER2 expression. Specifically, the core (NAcc) showed a highly rhythmic pattern of PER2 expression (R^2^ = 0.690; Fig. 8M–O) with amplitude of 176.78 (2nd highest after the SCN). Peak PER2 expression in this region occurs at approximately ZT22.5, 1-h earlier than the four subregions of the dorsal striatum. In contrast, as can be seen by the spread of data points in Fig. 8R, PER2 expression in the shell (NAcsh) was evenly distributed throughout the 24-h day. The extremely low amplitude of 4.33 and an R^2^ value of 0.006 did not meet the criteria for rhythmicity and, therefore, PER2 expression in this region was deemed arrhythmic under the current experimental conditions.

#### Striatum: Summary

PER2 expression patterns in the four subregions of the dorsal striatum are similar to one another, with identical time of peak expression and comparable amplitudes. None of the rhythms in these regions is in antiphase with the rhythm of the SCN. Moreover, the regions of the dorsal and ventral striatum differ slightly in phase as well as differ greatly in amplitude (SCN included: *F*_(6,554)_ = 109, *p*<0.0001, Fig. 6A). Specifically, the amplitude in the NAcc is higher than in all other striatal regions (ASd: *t*_(559)_ = 7.98, *p*<.001, ASm: *t*_(559)_ = 7.71, *p*<.001, PSd: *t*_(559)_ = 9.76, *p*<.001, PSm: *t*_(559)_ = 7.70, *p*<.001, NAcsh: *t*_(559)_ = 13.30, *p*<.001), whereas the amplitude in the NAcsh is lower than in all other striatal regions (ASd: *t*_(559)_ = 5.75, *p*<.001, ASm: *t*_(559)_ = 5.95, *p*<.001, PSd: *t*_(559)_ = 3.98, *p*<.01, PSm: *t*_(559)_ = 5.96, *p*<.001). Furthermore, the PER2 amplitude in the SCN is significantly higher than in all the striatal subregions. However, when the RI values are calculated, all regions of the striatum, except for the NAcsh, show moderate to strong rhythms with values ranging from 0.59 to 0.81 (Fig. 6B). The NAcsh shell, which we determined to be arrhythmic, has an RI value of 0.05 relative to the SCN, well below the 0.25 criteria level. Correlations between PER2 expression between the five rhythmic regions of the striatum and the SCN reveal statistically significant moderate to high relationships between these regions (see Table 4). Notably, the SCN is moderately negatively correlated with all sub-regions of the striatum, while the sub-regions of the striatum are highly positively correlated with each other, with r-values ranging from 0.70 to 0.91.

**Table 4:** Correlations of PER2 expression in the striatum.

Region	ASd	ASm	PSd	PSm	NAcc
SCN	−0.52	−0.47	−0.44	−0.45	−0.41
ASd		0.91	0.83	0.83	0.84
ASm			0.81	0.88	0.82
PSd				0.89	0.70
PSm					0.76

Pearson r values comparing regions of the striatum and the SCN across the 24-h day. Values in bold are statistically significant (*p*<0.05). The NAcsh has been omitted since it is arrhythmic.

### Cortex

#### PER2 expression in the endopiriform cortex, dorsal (EPd)

**Figure 9:**
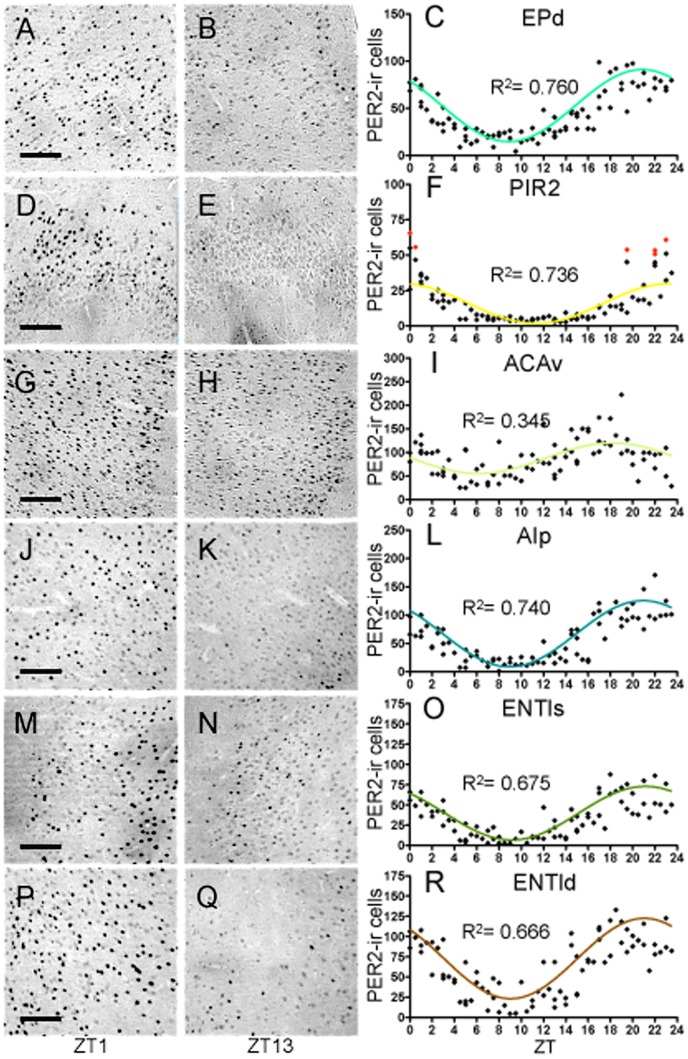
Cortex. Representative photomicrographs of PER2-ir cells in the endopiriform cortex, dorsal (**A–B**), piriform cortex, layer II (**D–E**), anterior cingulate area, ventral (**G–H**), agranular insular cortex, posterior part (**J–K**), entorhinal, lateral (ENTl), superficial layers (**M–N**), and ENTl, deep layers (**P–Q**) at ZT1 and ZT13, scale bar 100 µm. Mean number of PER2-ir cells in the EPd (**C**), PIR2 (**F**), ACAv (**I**), AIp (**L**), ENTls (**O**), and ENTld (**R**) of each individual rat (diamonds) across 48 *zeitgeber* times fitted with a 24-h sine wave. R^2^ = Goodness of fit value for given region. *n* = 84 for Epd, ACAv, and AIp, *n* = 78 for PIR2, *n* = 79 for ENTl deep and superficial layers (F: outliers = red diamonds).

The EP is described by some as layer IV of the PIR cortex due to its proximity and heavy interconnections [52]. Like the PIR, the EP not only has connections with the olfactory system, but also possesses reciprocal connections with the ENT, insular cortex, cortical amygdaloid nuclei, thalamus, and NAc shell [53]. As shown in Fig. 9C, there was a clear rhythmic pattern of PER2 expression (R^2^ = 0.760) in the EPd with amplitude of 76.22 and peak expression at ZT21.

#### PER2 expression in the piriform cortex, pyramidal layer II (PIR2)

The PIR is known as the primary olfactory cortex with major afferent input from the olfactory bulbs (OB). In addition to direct input from the OB, the PIR receives projections from the basal forebrain, thalamus, hypothalamus, and brainstem [54]. The PIR itself projects back to the OB, hypothalamus, and thalamus, and sends efferent projections to the insular cortex, ENT, and amygdala [54]. Besides playing a major role in olfactory processing, the PIR has also been implicated in fear memory [55] and epileptogenesis [54]. The PIR2 showed a rhythmic pattern of PER2 expression (R^2^ = 0.736) with low amplitude of 28.06 (Fig. 9D–F). Peak PER2 expression in the PIR2 occurred at ZT23.5, 2.5-h later than in the EPd.

#### PER2 expression in the anterior cingulate area, ventral (ACAv)

The ACA is important for a wide range of autonomic functions, including regulation of heart rate and blood pressure, and in motivation and goal-directed behaviors [56]. This cortical region can be subdivided anatomically and functionally into ventral (emotional) and dorsal (cognitive) divisions. The ventral ACA, studied here, has connections with the NAc core, amygdala, AI, and hypothalamus, and has been shown to be involved in the processing of salient emotional and motivational information [57], [58]. Furthermore, both the anterior and posterior cingulate project to the deep layers of the ENT [59]. The ACAv exhibited a relatively low rhythmic pattern of PER2 expression (R^2^ = 0.345) with amplitude of 64.66 (Fig. 9G–I). Peak PER2 expression occurred at approximately ZT18. However, the model failed the normality test (K^2^ = 6.41, *p*<0.05).

#### PER2 expression in agranular insular cortex, posterior (AIp)

The agranular insular (AI) cortex is part of the anterior portion of the insular cortex so named because of the missing granular layer IV [60]. The AI innervates the NAc core and shell, the BNST, and the amygdala, and itself receives large input from the CEAl as well as the ENTl and thalamus [61]–[63]. The AI also has reciprocal connections with the PIR and EP cortices. The insular cortex has been shown to be important in addiction, emotions, and motor control, as well as in homeostatic functions including visceral sensation and gustatory processing [60], [64]. In this area there was a highly rhythmic pattern of PER2 expression (R^2^ = 0.740) with amplitude of 116.74 and peak expression occurring at approximately ZT21 (Fig. 9J–L).

#### PER2 expression in the lateral entorhinal cortex (ENTl), superficial and deep layers

The ENT cortex is both the major input and, together with the CA1 and subiculum, the major output system of the hippocampal formation, projecting mainly to the amygdala, especially the BLA, and the NAc, and to other cortical areas such as the anterior and posterior cingulate cortex (reviewed in [65]). The six layers of the ENT have been classically divided into superficial (I–III) and deep (IV–VI) layers, with the superficial layers acting as the primary ‘input’ station and the deep layers acting as the primary ‘output’ station [46], [66], [67], although there is some dispute to whether this still holds true [68].

The superficial and deep layers of the ENTl showed similar patterns of PER2 expression (Fig. 9O & R, respectively). However, the deep layers had higher amplitude (99.2 vs. 66.14) and higher overall levels of PER2 compared to the superficial layers. The two subdivisions of the ENTl also had similar R^2^ values (0.675 for the superficial and 0.666 for the deep layers). Peak PER2 expression in the superficial layers occurred at approximately ZT21.5. The data for the superficial layers came from layers II and III of the ENTl, as there were no detectible PER2 labeled cells in layer I. Peak PER2 expression in the deep layers of the ENTl occurred slightly earlier, at approximately ZT21, in phase with two other regions of the cortex, the AIp and EPd.

#### Cortex: Summary

PER2 rhythms in the subregions of the cortex studied differ in phase of peak expression. Specifically, PER2 expression in the ENTls peaks 30 min later than in the ENTld, EPd, and AIP (which are all in phase); expression in the PIR2 peaks almost 3-h later, whereas the rhythm of PER2 expression in the ACAv peaks 4-h earlier than in the ENTld/EPd/AIp cluster. Once again, none of the rhythms in these regions is in phase (or anti-phase) with the rhythm in the SCN. Furthermore, PER2 rhythms in the cortical regions studied differ in amplitude (SCN included: *F*_(6,562)_ = 128.90, *p*<0.0001). Specifically, the amplitude in the PIR2 is significantly lower than in all other cortical regions (EPd: *t*_(567)_ = 4.67, *p*<.001, ACAv: *t*_(567)_ = 3.55, *p*<.01, AIp: *t*_(567)_ = 8.59, *p*<.001, ENTls: *t*_(567)_ = 3.63, *p*<.01, ENTld: *t*_(567)_ = 6.79, *p*<.001), and the amplitude in the AIp is higher than in the EPd (*t*_(567)_ = 4.00, *p*<.01), ACAv (*t*_(567)_ = 5.14, *p*<.001), and ENTls (*t*_(567)_ = 4.92, *p*<.001, Fig. 6A). Finally, the amplitude in the SCN is significantly higher than in all the cortical regions. However, when RI values for these regions are normalized to the SCN, it becomes evident that most of the sub-regions of the cortex show strong rhythms (Fig. 6B). Specifically, the EPd has a rhythm that is 72% as rhythmic as the SCN, while surprisingly, the cortical region with the lowest amplitude, the PIR2, has a very strong rhythm comparable to that of the SCN (RI value of 0.89). The ACAv has the weakest rhythm out of the cortical regions with a rhythm that is under half as rhythmic as that of the SCN (RI value of 0.37), while the AIp is 87% as rhythmic as the SCN. Finally, the two regions of the ENTl cortex show differences in rhythmicity levels with the ENTls having a slightly stronger rhythm than the ENTld (RI values of 0.83 and 0.68, respectively). Correlations of PER2 expression across the 24-h day between the six regions of the cortex and the SCN reveal differences in the strength of the relationships between these regions (see Table 5). Notably, the SCN is only significantly correlated with two sub-regions of the cortex, the ACAv (0.43) and PIR2 (−0.56). Not surprisingly, the three regions that are in perfect phase, the EPd, AIp, and ENTld, are all highly correlated with each other, with r-values ranging from 0.84 to 0.86.

**Table 5:** Correlations of PER2 expression in the cortex.

Region	ACAv	AIp	EPd	PIR2	ENTls	ENTld
**SCN**	**0.43**	**−**0.11	**−**0.07	**−0.56**	**−**0.07	**−**0.06
**ACAv**		**0.38**	**0.46**	0.11	**0.50**	**0.55**
**AIp**			**0.86**	**0.67**	**0.88**	**0.84**
**EPd**				**0.67**	**0.86**	**0.84**
**PIR2**					**0.68**	**0.64**
**ENTls**						**0.91**

Pearson r values comparing regions of the cortex and the SCN across the 24-h day. Values in bold are statistically significant (*p*<0.05).

### PER2 Expression: Summary

**Figure 10:**
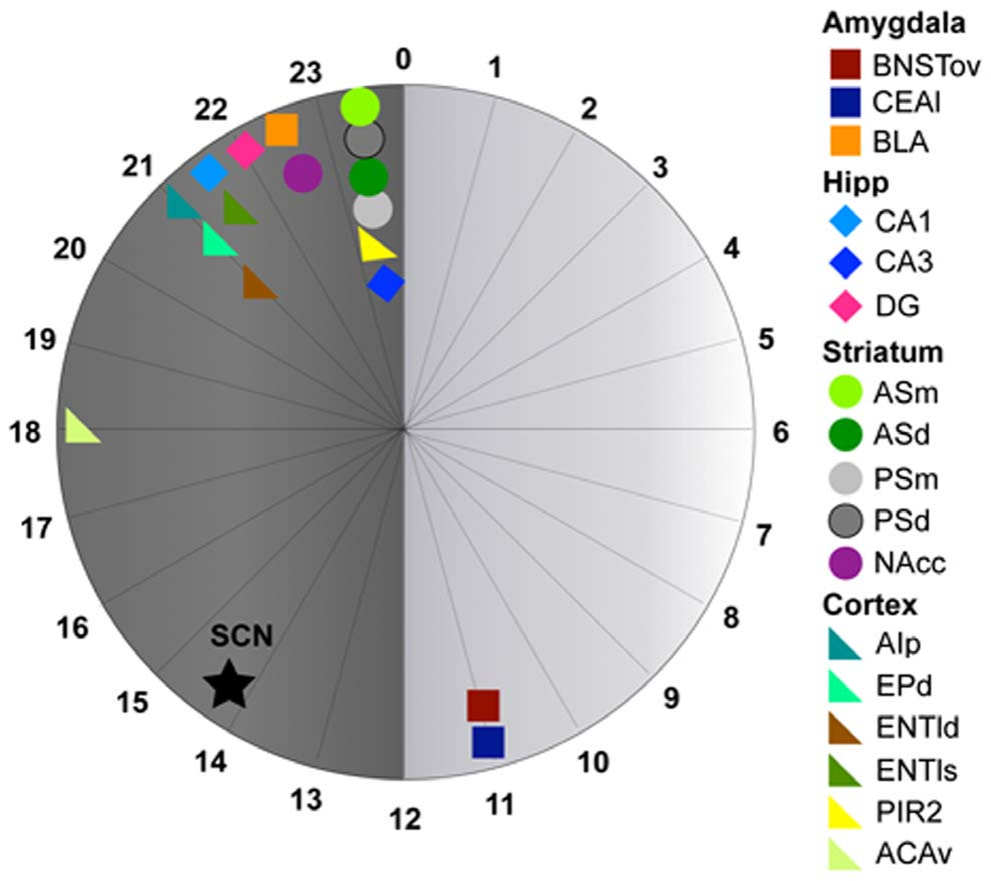
Clustered phases of peak PER2 expression. Twenty-four hour circular diagram displaying peak PER2 expression in all 18 rhythmic regions analyzed. Numbers around the ‘clock’ are in ZT time. 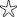: SCN, □: amygdala, ⋄: hippocampus, ○: striatum, 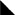: cortex.

The high temporal resolution analysis used in this study revealed that the pattern and timing of PER2 expression varied considerably between brain regions. Although most regions studied exhibited daily rhythms of PER2 expression, two regions, the MEApd and NAcsh, were found to be arrhythmic. The regions studied can be grouped into four “clusters” based on time of peak PER2 expression, with a cluster being defined as multiple regions peaking within 30 min of each other (Fig. 10). Notably, whereas in most regions PER2 expression peaked during the dark phase (between ZT12–ZT24), expression in the BNSTov and CEAl peaked towards the end of the light phase at ZT11. Four regions of the cortex (the AIp, EPd, ENTl deep and superficial) and the CA3 formed a cluster around ZT21–ZT21.5; the BLA and NAc core formed a cluster at ZT22.5; whereas the DG peaked in the middle of these two clusters 30 min from each at ZT22. The four subregions of the dorsal striatum along with the CA3 and PIR2 formed a cluster at ZT23.5. The peaks of the rhythms of PER2 expression in the SCN and ACAv differed both from each other and from all other regions studied. Finally, it was found that regardless of their low amplitude (Fig. 6A), most of the regions analyzed had strong rhythms, as determined by the RI (Fig. 6B).

## Discussion

We used a high temporal resolution analysis to characterize the distribution, amplitude and phase of PER2 rhythms in the rat forebrain. Of the 20 PER2 expressing areas studied, 18 showed measurable daily variations in PER2 expression. Analysis of these rhythms further revealed marked regional differences in amplitude and phase of peak expression. Our results show that for most forebrain regions studied PER2 expression peaks during the last quarter of the dark portion of the LD cycle. In contrast, PER2 expression in the BNSTov and CEA peaks just before the end of the light phase, and in the ACAv, PER2 peaks in the middle of the night. PER2 expression in the SCN peaks at a unique phase, shortly after the onset of the dark portion of the cycle. The results underscore the complex organization of the central circadian system and support the view that a distributed network of differently phased, rhythmic brain structures mediates circadian, and potentially other, behaviors in rats.

Although the presence of clock genes such as *Per1*, *Per2*, and *Clock* in several brain regions has previously been reported [69], [70], this is the first *in vivo* high temporal resolution analysis of PER2 rhythms performed in a rodent. The present results expand earlier observations of circadian oscillations in some extra-SCN regions using electrophysiological recording and bioluminescence imaging techniques [8], [10], [71], [72] and refine and expand upon our previous findings concerning PER2 rhythms in various forebrain regions [13], [15], [19], [73]. Specifically, the present analysis points to a complex organization of PER2 rhythms in the forebrain, with oscillations in different regions falling into at least four distinct phase clusters. Intriguingly, in some cases, PER2 rhythms in interconnected regions within the same broader brain structure were found to belong to different phase clusters. For example, in the hippocampus, PER2 expression in the CA3 peaks 1.5–2 hours later than in the DG and CA1, and in the amygdala, PER2 expression in the CEA peaks almost in antiphase with that of the BLA.

Two out of the 20 sub-regions analyzed, the NAc shell and the posteriodorsal part of the medial amygdala (MEApd), failed to meet the criteria for rhythmicity. Both areas expressed PER2 immunoreactivity, however no significant time of day variations was noted leading to the classification of these regions as arrhythmic. Lack of tissue rhythmicity might suggest that individual PER2 expressing cells within these regions are themselves arrhythmic [74]. Alternatively, individual neurons in the NAc shell and MEApd might be rhythmic but are not synchronized to each other, resulting in overall arrhythmicity. The latter possibility is consistent with studies in the SCN where it was found that individual cells within an arrhythmic SCN still oscillate with a period close to 24-h but are out of phase with each other [75]. Although we found that under normal conditions PER2 expression in the NAc shell and MEApd is arrhythmic, it is possible that a rhythm could be induced in these regions under specific experimental conditions, as has been shown to occur in the dorsomedial hypothalamus (DMH) following exposure to a restricted feeding regimen [23], [76], [77]. Given the important role of the NAc shell in drug addiction, it would be interesting to see if a rhythm in PER2 could be induced in this region by timed, daily drug administration.

### Functional Role of Multi-oscillator Organization

The mammalian circadian system is viewed as a hierarchical network in which a master circadian clock located in the SCN synchronizes scattered populations of central and peripheral cellular oscillators that control tissue specific outputs. Importantly, in spite of the similarity in molecular composition, the rhythm of the SCN differs from those in other central and peripheral tissues. Whereas in the SCN the rhythms are intrinsically synchronous and self-sustaining, rhythms in most other tissues rely on synchronizing input from the SCN communicated via neural projections and diffusible factors as well as through autonomic and endocrine pathways and behaviors under SCN control [2]–[4]. The present results support the view that the central circadian system is comprised of multiple, brain region-specific clocks “chiming” at different times of day, and are consistent with the notion that this complex temporal organization is important for optimal adaptation to subtle changes in the environment, such as alterations in seasonal day length [78]. Notably, however, this temporal organization can be disrupted by abrupt, large changes in environmental timing cues, such as those induced by travel across time zones and rotating shift work. In agreement with this, rodents experiencing large shifts in the LD cycle display temporary disruption of the normal phase relationship between the rhythms of the SCN and those of cellular clocks throughout the rest of the body. This disruption lasts for several days before the normal phase relationship is re-established following re-entrainment to the new LD cycle. For example, whereas *Per1* rhythms in the SCN shift rapidly following a 6-h phase shift of the LD cycle, clocks in peripheral tissues and brain structures take significantly longer (up to 6 days) to re-entrain [78], [79]. Our laboratory has shown that re-entrainment of the PER2 rhythm in the BSNTov to an 8-h delay or advance in the LD cycle lagged behind the SCN by several days before re-establishing its normal phase relationship [13]. Furthermore, we have shown that prolonged exposure to a 26-h LD cycle, which requires daily phase delays, uncouples PER2 rhythms in the BNSTov and CEAl from the rhythm of the SCN, while leaving rhythms in other limbic forebrain regions unaffected, and leading to the emergence of a new phase relationship between these regions [80].

It is believed that one of the key roles of circadian oscillators in the brain is to maintain the operational integrity of neural circuits by regulating basic processes at the cell and tissue levels [81]. Accordingly, changes in the timing of such processes within multiple brain areas may affect the functioning of these circuits and, ultimately, alter behavior. Consistent with this idea, disruptions in circadian rhythms have been associated with sleep disturbances, cognitive dysfunction, mood disorders, and general malaise as seen in jet lag and shift work [82]–[84].

The present study provides a snapshot of PER2 rhythms under basal conditions in brain regions known to play important roles in a wide range of motivated and appetitive behaviors. The fact that PER2 rhythms in multiple regions exhibit differential patterns in peak expression suggests that it is the phase relationship between the rhythms in these regions that establishes a healthy functioning system. Importantly, although the SCN is the primary synchronizer of rhythmicity, several other factors have been identified that can directly affect the phase of PER2 rhythms in different regions of the brain, including homeostatic and hormonal perturbations that can directly influence behavior. Most notably, restricted feeding schedules are able to entrain or shift rhythms in multiple nuclei, effectively overriding SCN signals [76], [85]–[87]. PER2 rhythms in the BNSTov and CEAl have been shown to be selectively sensitive to rhythmic corticosterone [88], thyroid hormones [73], and gonadal hormones [22] while other regions in the limbic forebrain remain unaffected [89], [90]. The neurotransmitter dopamine (DA) was found to be involved in the regulation of PER2 protein and *Per2* mRNA in the dorsal striatum [14]. Interestingly, the effect of DA on PER2 rhythms in the dorsal striatum was found to be mediated by the D_2_ DA receptor [14], and preliminary work in our laboratory shows that PER2 is co-localized within neurons expressing DA D_2_ receptors or enkephalin in the dorsal striatum [91]. It is important to note that since the rats in this experiment were entrained to a LD cycle, it is unknown whether the PER2 rhythms in the regions examined are endogenous or a result of this zeitgeber. Understanding the nature of the signals that modulate clock gene oscillations observed in extra-SCN regions will provide a critical starting point for the understanding of the role of these clocks in normal and pathological conditions.

### Conclusion

Our data demonstrate the presence of complex and previously unappreciated differences in rhythms of PER2 expression between functionally and anatomically interconnected brain regions, as well as between these brain areas and the master SCN clock. The range of phases of peak expression observed in different brain areas likely represents an optimal organization required for a flexible multi-oscillatory system capable of being reset daily by environmental cues.
